# Further developments towards a genome-scale metabolic model of yeast

**DOI:** 10.1186/1752-0509-4-145

**Published:** 2010-10-28

**Authors:** Paul D Dobson, Kieran Smallbone, Daniel Jameson, Evangelos Simeonidis, Karin Lanthaler, Pınar Pir, Chuan Lu, Neil Swainston, Warwick B Dunn, Paul Fisher, Duncan Hull, Marie Brown, Olusegun Oshota, Natalie J Stanford, Douglas B Kell, Ross D King, Stephen G Oliver, Robert D Stevens, Pedro Mendes

**Affiliations:** 1 School of Chemistry, The University of Manchester, Manchester M13 9PL, UK; 2 Manchester Centre for Integrative Systems Biology, The University of Manchester, 131 Princess Street, Manchester, M1 7DN, UK; 3 School of Mathematics, The University of Manchester, Oxford Road, Manchester M13 9PL, UK; 4 School of Computer Science, Kilburn Building, The University of Manchester, Oxford Road, Manchester M13 9PL, UK; 5 School of Chemical Engineering and Analytical Science, The University of Manchester, Oxford Road, Manchester M13 9PL, UK; 6 Cambridge Systems Biology Centre & Department of Biochemistry, University of Cambridge, 80 Tennis Court Road, Cambridge CB2 1GA, UK; 7 Department of Computer Science, Aberystwyth University, SY23 3DB, UK; 8 Doctoral Training Centre for Integrative Systems Biology, The University of Manchester; 9 Virginia Bioinformatics Institute, Virginia Tech, Washington Street 0477, Virginia 24061, USA

## Abstract

**Background:**

To date, several genome-scale network reconstructions have been used to describe the metabolism of the yeast *Saccharomyces cerevisiae*, each differing in scope and content. The recent community-driven reconstruction, while rigorously evidenced and well annotated, under-represented metabolite transport, lipid metabolism and other pathways, and was not amenable to constraint-based analyses because of lack of pathway connectivity.

**Results:**

We have expanded the yeast network reconstruction to incorporate many new reactions from the literature and represented these in a well-annotated and standards-compliant manner. The new reconstruction comprises 1102 unique metabolic reactions involving 924 unique metabolites - significantly larger in scope than any previous reconstruction. The representation of lipid metabolism in particular has improved, with 234 out of 268 enzymes linked to lipid metabolism now present in at least one reaction. Connectivity is emphatically improved, with more than 90% of metabolites now reachable from the growth medium constituents. The present updates allow constraint-based analyses to be performed; viability predictions of single knockouts are comparable to results from *in vivo *experiments and to those of previous reconstructions.

**Conclusions:**

We report the development of the most complete reconstruction of yeast metabolism to date that is based upon reliable literature evidence and richly annotated according to MIRIAM standards. The reconstruction is available in the Systems Biology Markup Language (SBML) and via a publicly accessible database http://www.comp-sys-bio.org/yeastnet/.

## Background

A central goal of integrative systems biology is the accurate representation of molecular interaction networks. Ultimately, such networks can be used to underpin mathematical models, consisting of stochastic or ordinary differential equations that permit the simulation of biological behaviour. The first step in generating such models is constructing a network of biochemical reactions and interactions between molecular components of the system to form a qualitative (unparameterised) model. Several groups have reconstructed the metabolic network of baker's yeast from genomic and literature data [1-3]. Variation in the approaches used, and contradictory interpretations of the available literature, mean that most reconstructions differ considerably. To resolve these problems, a cohort of the yeast systems biology community collaborated to create a consensus reconstruction. In April 2007, a large focused meeting brought together experts from various groups and disciplines in order to resolve discrepancies between the various reactions and metabolites described by other available reconstructions and form a consensus. The resultant reconstruction [4], subsequently referred to as "Yeast 1.0", removed the ambiguities inherent in its predecessors through the use of principled and computer-readable annotations. Whilst previous reconstructions had defined entities using subjective names, which lacked precision and resulted in ambiguities, Yeast 1.0 directly referenced chemical and protein descriptions to persistent databases or used standardised, database-independent, computer-readable representations. This removed the ambiguities and allowed the new reconstruction to be used effectively as the basis for automated analyses.

A limitation of Yeast 1.0 came about through the very generation of the consensus; the network became considerably fragmented as reactions that could not be readily annotated (due to the presence of structural ambiguities) were removed. This led to underrepresentation of a number of pathways, particularly those involved in lipid biosynthesis. Since Yeast 1.0, many improvements have been made to the reconstruction. The latest release, described here, is considerably larger (in terms of numbers of metabolites and reactions), of higher quality (by reference to literature evidence), exhibits greater coverage of known metabolic enzymes, and is better connected than all previous efforts.

**Figure 1:**
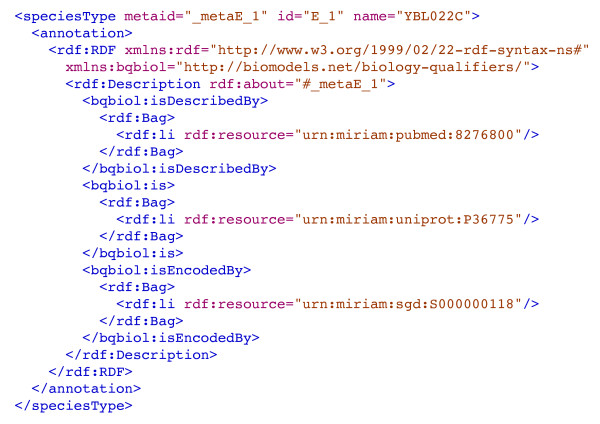
**SBML example**. Simplified example of MIRIAM-compliant SBML, whereby an enzyme is annotated with reference to the databases UniProt, SGD and PubMed, respectively.

The reconstruction is described and made available in Systems Biology Markup Language (SBML) [5], an established community XML format for the mark-up of biochemical models. With the introduction of SBML Level 2, specific model entities, such as species or reactions, can be annotated using ontological terms. These annotations, encoded using the resource description framework (RDF) [6], provide the facility to assign definitive terms to individual components, allowing the software to identify such components unambiguously and thus link model components to existing data resources [7]. Minimum Information Requested in the Annotation of Models (MIRIAM) [8] -compliant annotations have been used to identify components unambiguously by associating them with one or more terms from publicly available databases registered in MIRIAM Resources [9]. An example of such an annotation is presented in Figure 1, where an enzyme is identified by MIRIAM-compliant references to the UniProt [10], SGD [11], and PubMed [12] databases. Metabolites are annotated with reference to the ChEBI (Chemical Entities of Biological Interest) database [13]. Whilst SBML is the primary format for dissemination of the reconstruction, we also make the reconstruction available in an online database [14], B-Net, that enables easy searching of the content. B-Net [15] is able to represent all of the SBML features utilised in the current reconstruction. Searches can be performed using synonyms and the user is also able to navigate through the network from any point (e.g. a metabolite, reaction or enzyme) to its connected neighbours. Query results can also be exported in SBML and this is an effective mechanism to extract subsets of the entire model in this exchange format.

## Results and Discussion

Improvements in the representation of yeast metabolism in this release as compared to Yeast 1.0 primarily consist of its enhanced representation of lipid metabolism and greater connectivity, thereby permitting constraint-based flux analyses. Many of the extensions to Yeast 1.0 are reactions garnered from the literature, which are entirely novel to any genome-wide yeast metabolic reconstruction. Data were also incorporated, when backed up by traceable evidence, from two other reconstructions: iMM904 [16] and iIN800 [17]. The resulting consensus network (reported in Additional File 1) consists, in decompartmentalised form, of 1102 metabolic reactions involving 924 metabolites and 924 proteins (Table 1) and is therewith larger in scope than any previous reconstruction.

**Table 1:** Reconstruction scope

	iMM904	iIN800	Yeast 1.0	Yeast 4.0	change (%)
**reactions**	1050	907	962	1102	14.6
**metabolites**	872	812	813	924	13.7
**proteins**	904	707	832	924	11.0
**compartments**	8	4	15	16	6.7

Comparison of the scope of reconstructions (Yeast 4.0 being the version number of the current reconstruction). Metabolites and reactions in different intracellular compartments are considered one, as are reactions with the same stoichiometry (isoenzymatic). This renders reconstructions with differing granularity comparable.

**Figure 2:**
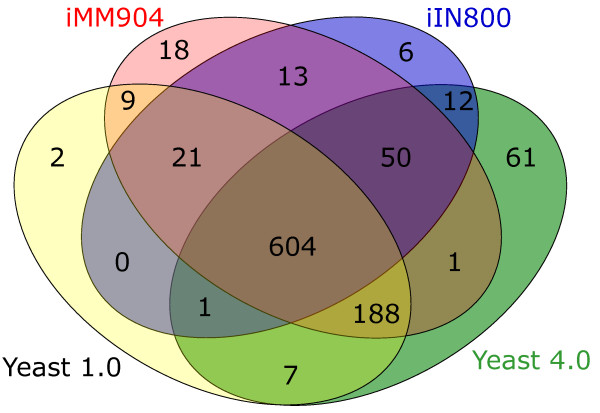
**Comparison of reconstructions in terms of enzymes present**. The reconstruction presented here contains 124 more enzymes than Yeast 1.0, 61 of which have not been considered by any of the other reconstructions. Yeast 1.0 was also improved upon through better curation leading to the removal of (2 + 9 + 21 =) 32 enzymes. A further (6 + 13 + 18 =) 37 enzymes from iMM908 and iIM800 were not added to the reconstruction.

Careful curation does not simply involve increasing the scope of the reconstruction. Indeed, 32 enzymes from Yeast 1.0 were considered insufficiently evidenced and have been removed, whilst a number of metabolites were relocalised to a different compartment. A typical example of an enzyme removed from the reconstruction is Gpm2p; whilst a homologue of Gpm1p, its phosphoglycerate mutase activity could not be evidenced and may be non-functional [18]. Four reconstructions are compared in Figure 2 in terms of enzymes present. In addition to the 32 enzymes removed, the reactions of a further 37 enzymes from iMM904 and iIN800 have not been added for lack of supporting evidence. In total, the new reconstruction considers 124 more enzymes than its predecessor, with half of these (61) being retrieved manually from the literature and therefore new to all reconstructions.

### Lipid metabolism

The correct and complete representation of lipid metabolism is important, not only to meet the ultimate goal of genome-scale coverage, but also because understanding and engineering lipid metabolism through systems and synthetic biology is likely to play a major role in the replacement of fossil energy sources and chemical feedstocks with biofuels and bioplastics [19]. In Yeast 1.0, lipid metabolism was poorly captured. To move towards a better representation, the literature, database annotations and homology relationships were used to identify the set of lipid-related yeast enzymes. Homology with mouse and human enzymes reported in LipidMaps [20], and with enzymes from all organisms reported in KEGG lipid pathways [21], indicated lipid enzymes in yeast (homology relationships predefined by Ensembl [22]). Further enzymes were added to the set manually by examination of SGD and Ensembl annotations. A total of 268 yeast enzymes were identified as likely to be part of lipid metabolism. Although the boundaries of this set are unavoidably subjective, it appears to capture the majority of lipid-related genes in yeast.

**Figure 3:**
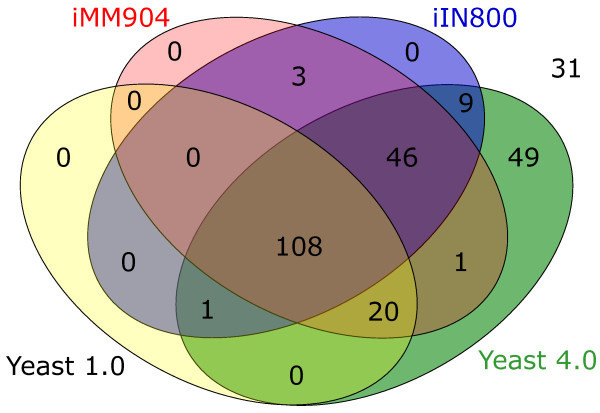
**Comparison of the coverage of lipid metabolism enzymes by the different reconstructions**. At least one reaction in a reconstruction is catalyzed by each enzyme. On top of extending Yeast 1.0 by (1 + 9 + 46 =) 56 enzymes from iMM904 and iIN800, a further 49 enzymes uniquely appear in this latest reconstruction. Three reactions common to iMM904 and iIN800, plus 31 others, have not been incorporated for lack of evidence.

With reference to this set of lipid enzymes, the iIN800 reconstruction of Nookaew *et al*. improved upon the original community reconstruction (Yeast 1.0) by increasing set coverage from 48% to 62% (with at least one reaction being associated with each enzyme). In the present release set coverage has further improved to 87%. Coverage of the lipid enzyme set by the various reconstructions is summarised in Figure 3. From iIN800 and iMM904, 56 lipid enzymes were added to Yeast 1.0, while three enzymes from these sources were not added. The current reconstruction describes activities for 49 enzymes that no other reconstruction has ever considered. Combining these, the reconstruction extends the Yeast 1.0 description of lipid metabolism by a total of 105 new enzymes, extends iMM904 by 59 enzymes, and iIN800 by 70 enzymes. This is by far the most comprehensive reconstruction of yeast lipid metabolism to date.

The 34 remaining lipid enzymes (in figure 3 these are 31 not found in any reconstruction, plus three found in both iMM904 and iIN800) from the set are either too poorly characterised functionally to be included or cannot be represented within the current description of the cell's compartmentalisation. Flippases, for example, require a more detailed description of membrane faces to capture their role in membrane asymmetry. Improving compartmental representation will be a goal for future releases.

### Connectivity

**Figure 4:**
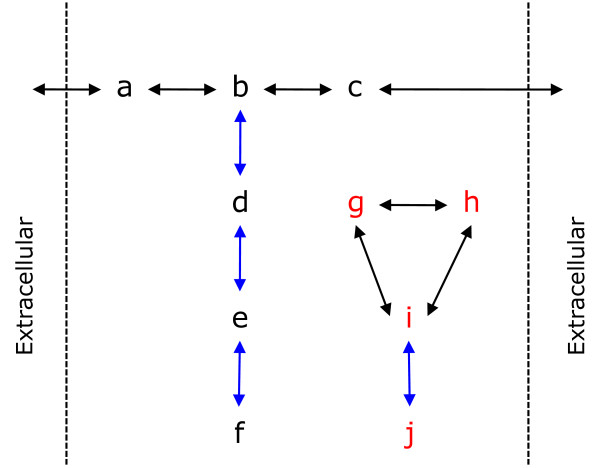
**Visualisation of connectivity analysis**. Metabolites that are unreachable (in red) were identified with a graphical analysis, by locating metabolites that are disconnected from the extracellular medium. Flux variability analysis identified reactions that must have zero flux (in blue) because they lead to dead-end metabolites.

Structural improvement was a major focus of the advancements made to the reconstruction by identifying and rectifying unconnected regions of the network. Two measures were used to describe connectivity. First, we identified clusters of unreachable metabolites; that is, clusters of metabolites that are disconnected from the extracellular medium, in a graph-theoretic sense, and thus cannot ever be produced by the reaction network. Secondly, we used flux variability analysis [23] to identify reactions that, by mass balancing, must have zero flux, for example because of dead-end metabolites (products that are not the substrates of another reaction). Led by these analyses, which are explained graphically in Figure 4, we looked for literature evidence describing these missing elements of our network. By targeting unreachable clusters and those reactions whose reconnection has the most influence on the network's connectivity, we maximised the impact of literature curation on modelling. By both measures, the present release improves both upon the previous release and particularly upon iMM904 and iIN800 (Table 2). More than 90% of metabolites can be reached from the extracellular medium and only 12.7% of reactions must have zero flux.

**Table 2:** Network connectivity

	iMM904	iIN800	Yeast 1.0	Yeast 4.0
**intracellular metabolites**	708	681	658	758
**unreachable**	440	468	108	75
**%**	62.2	68.7	16.4	9.9

**metabolic reactions**	1050	907	962	1102
**zero flux**	225	282	153	140
**%**	21.4	31.1	15.9	12.7

As in Table 1, decompartmentalised models were used to generate these data.

Our approach towards structural improvement is also an example of the iterative "cycle of knowledge" approach [24], where the model is first used to guide biological research and can subsequently be updated and improved as specific new knowledge becomes available. In this case the iteration consisted of discovery and collation of experimental evidence previously obtained but which had never been identified in this context. Such discovery of knowledge was informed by the previous models and was unlikely to have happened in their absence.

### Constraint-based analysis

New reconstructions are often validated through constraint-based approaches like Flux Balance Analysis (FBA) [25] to assess their ability to predict experimental results. While there is clear utility in deploying such methods to explore biochemical capacity, using improved agreement with experimental observations to determine whether the reconstruction is, in some sense, 'better' than previous efforts is potentially misleading. In the current release, non-inferred reactions are supported by evidence from the literature and it is in this sense that the reconstruction is validated and improved. That said, the updates improved the connectivity considerably and together with the inclusion of a reaction describing biomass composition now allows FBA to be performed. The availability of the model in SBML means that it is accessible through many generic and systems-biology-specific software packages, including the COBRA (COnstraint-Based Reconstruction and Analysis) toolbox [26].

The model was used to predict single knockout viability through flux balance analysis (FBA). Growth conditions exactly followed those set out in iMM904, namely a glucose-limited minimal medium. Cellular biomass was defined as in iIN800 (carbon-limited version), due to its high level of detail regarding lipid composition. As the reaction producing biomass does not represent a real metabolic process it is semantically annotated as such using SBO (Systems Biology Ontology) [27] identifiers and GO (Gene Ontology) [28] evidence codes to ensure this distinction is maintained (therefore allowing one to easily remove this reaction based on its annotation). Simulations were performed using COBRA (which is reliant on libSBML [29] and the GNU linear programming kit [30]). The simulation predictions were compared to a list of lethal gene knockouts. This list was generated by considering results from viability experiments under both rich [31] and glucose minimal growth medium conditions [32]. Results demonstrate similar performance to that of previous reconstructions in terms of the accuracy of prediction of single gene knockout viability (Table 3).

**Table 3:** Gene knockout analysis

	iMM904	iIN800	Yeast 4.0
**number of genes**	904	707	924
**true positive (%)**	75.0	69.7	74.8
**true negative (%)**	5.1	6.9	5.3
**false positive (%)**	9.3	10.6	11.1
**false negative (%)**	10.6	12.7	8.8

Results of *in silico *viability prediction of deletion strains of *S. cerevisiae*. "Positive" and "negative" refer to the ability and inability to grow, respectively. Following earlier studies, the knockout simulation was conducted in a glucose-limited minimal medium, and compared to experimental knockout data [30,31].

Closer inspection of predictions reveals that relatively subtle network variations often underlie prediction differences. Four experimentally lethal knockouts were not initially predicted as such by the new reconstruction, but are correctly predicted using iMM904. Three of these genes encode enzymes that are essential to riboflavin biosynthesis. The capacity of iMM904 to predict lethality correctly is due to its biomass definition including a small contribution from riboflavin, whereas this was not part of the initial iIN800 or current network's biomass definition. Subsequent addition of riboflavin to the (empirical) biomass description has resolved these differences. Note that this is not therefore a reflection of the quality of the underlying network but only of the empirical biomass estimation, which is itself dependent on the growth conditions.

In places, the added richness of the new reconstruction combines with certain known limitations to defeat total agreement with experiment. An example is seen by knocking out the *acs2 *gene, encoding acetyl-coA synthetase (Acs2p). By experiment this should be lethal, yet in the current network the cytoplasmic reaction is also catalysed by Acs1p, consistent with experimental data [33]. When the Acs2p-catalysed reaction is eliminated, flux simply re-routes through the Acs1p reaction. Importantly, it is only the fortuitous incompleteness of iMM904, lacking the cytosolic Acs1 isozyme that reveals the inviability of the *acs2 *knockout. The proper basis of the inviability of the *acs2 *mutant is that *ACS1 *is transcriptionally repressed in the high glucose conditions of viability experiments and so is unable to compensate for the loss of *ACS2 *[34]. Transcriptional control is not captured in the metabolic network and thus cannot be captured in metabolic reconstructions of this type.

Both these examples highlight the caution required when using approaches such as FBA to validate reconstructions. The added detail in the present network can naturally lead to an increase in false positive outcomes: *in silico *knockouts that are overcome by alternative routings in the network but are actually lethal *in vivo*. This is, however, tempered by a decrease in false negative outcomes (i.e. knockouts that appear lethal computationally but are viable *in vivo*, as presented in Table 3).

### Uncharacterised enzymes

Despite the much-increased coverage of the current reconstruction, 451 genes probably encode metabolic enzymes that still have no associated reaction (Additional file 2). For the majority of these, very little is known about their function and further characterisation is required. From the viewpoint of furthering systems biology reconstruction efforts, these enzymes are important targets for reductionist molecular biology studies, including, for instance, systematic analyses using the Robot Scientist approach [35]. Their listing here is a motivation for further iterations on the cycle of knowledge.

## Conclusions

The development of high quality, well annotated, genome-scale, metabolic networks is an ambitious, challenging, but necessary step towards the realisation of integrative systems biology. While networks predicted through bioinformatics approaches are useful, particularly for the extension of systems biology approaches to less well-studied organisms, reconstructions built upon solid biochemical evidence provide a gold standard upon which predictions can be reliably based. For metabolic reconstructions, where the goal is to capture maximally our current understanding of metabolism, these problems are primarily of data integration and quality. It has proven essential to involve the extended systems biology and yeast communities in this process, both to establish the mechanisms and structures for acquiring and representing information, and also to tap into expert knowledge from the various sub-disciplines of biology and biochemistry. In the recent very large-scale reconstruction of the yeast molecular interaction network by Aho *et al*. [36], genomic, transcriptomic, proteomic and metabolomic data were integrated. These authors note that incorporating the higher quality data of Yeast 1.0 (and therefore even more of this contribution) would considerably improve their reconstruction over the metabolic information extracted from KEGG, and also that standards compliance is essential to this integration task.

Yeast 1.0 set standards and amalgamated existing networks, enhancing annotation and removing less reliable data. In this latest reconstruction, we have made significant headway on the process of filling gaps in the network. There is still some way to go before realising the goal of at least one reaction for each putative metabolic enzyme and, if one also considers enzyme promiscuity [37,38], even this will represent an incomplete picture of metabolism. This latest reconstruction is a considerable improvement on previous releases, particularly in describing lipid metabolism and addressing gaps in the original reconstruction that hindered modelling efforts. Information from other reconstructions since Yeast 1.0 has been incorporated, although not indiscriminately, and very many reactions not found in other reconstructions have been garnered from the literature. It is considerably larger than all previous efforts, while maintaining compliance with community-defined standards.

While Yeast 1.0 represented a major advance, particularly through the definition of standards and by the involvement of the wider yeast community, a major flaw was that it was not amenable to constraint-based analysis. The current reconstruction rectifies this, mostly by filling in gaps but also by inclusion of an appropriately annotated "biomass" reaction, without compromising the strict evidence requirements of its predecessor. When compared to experimental knockout data, this reconstruction did not identify certain lethal knockouts that other yeast reconstructions correctly predicted, but proves better than them in recognising viable deletions. This is a direct result of the richness of the model; as with the example of the acetyl-coA synthetases (above), addition of isoenzymes of specific reactions that do not exist in earlier reconstructions can reduce the predictive power of the model. Nonetheless, such enzymes are included due to literature support. This reconstruction continues the shifting focus, started with the consensus model Yeast 1.0, toward realistic representation and proof-based selection of reactions, rather than creating a reconstruction with simulation in mind. Reactions with a lower level of confidence (e.g. biomass definition) are characterised with specialised evidence codes and SBO terms, allowing the easy extraction of subsets of the network from the SBML code for specific purposes.

To facilitate further improvements, we encourage the community to provide information and/or corrections to the current release. We have set up a dedicated point-of-contact to this end network.reconstruction@manchester.ac.uk. We also highlight gaps in the network that cannot be resolved from current literature, as well as the little-studied enzymes for which we have not yet identified any function (see Additional File 2). These represent potentially important research opportunities for the community and we welcome efforts towards an improved understanding of their functions.

## Authors' contributions

PDD, KS, DJ, ES, KL, PP, NS, WBD, DH, MB, OO, NJS and PM contributed to literature curation to identify new reactions. KS and NS prepared and curated the SBML. PF collated relevant literature for curation. PDD, KS, DJ, ES, DBK and PM wrote the manuscript. CL, DBK, RDK, SGO, RDS and PM supervised work and/or contributed to discussions. All authors read, improved, and approved the final manuscript.

## Supplementary Material

Additional file 1 **Yeast SBML files**. ZIP file containing the latest reconstruction in SBML format. The metabolic network reconstruction is described using MIRIAM-compliant SBML, compatible with many Systems Biology software packages, including the COBRA toolbox. The model is also available in decompartmentalised form, and in an old SBML format (level 2, version 1) for backward compatibility. Click here for file

Additional file 2 **Poorly characterised genes**. Excel spreadsheet. The network is built upon intensive literature mining to identify reactions. Many genes still do not have detailed literature describing the functions of their products, yet (by what little is known or through sequence analysis) they appear likely to be involved in metabolism. The attached list describes these genes. Click here for file

## References

[B1] Förster J Famili I Fu P Palsson BØ Nielsen J Genome-scale reconstruction of the Saccharomyces cerevisiae metabolic network Genome Research 2003 13 2 244 253 10.1101/gr.234503 12566402 PMC420374

[B2] Duarte NC Herrgård MJ Palsson BØ Reconstruction and validation of Saccharomyces cerevisiae iND750, a fully compartmentalized genome-scale metabolic model Genome Research 2004 14 7 1298 1309 10.1101/gr.2250904 15197165 PMC442145

[B3] Kuepfer L Sauer U Blank LM Metabolic functions of duplicate genes in Saccharomyces cerevisiae Genome Research 2005 15 10 1421 1430 10.1101/gr.3992505 16204195 PMC1240085

[B4] Herrgård MJ Swainston N Dobson P Dunn WB Arga KY Arvas M Blüthgen N Borger S Costenoble R Heinemann M A consensus yeast metabolic network reconstruction obtained from a community approach to systems biology Nature Biotechnology 2008 26 10 1155 1160 10.1038/nbt1492 18846089 PMC4018421

[B5] Hucka M Finney A Sauro HM Bolouri H Doyle JC Kitano H Arkin AP Bornstein BJ Bray D Cornish-Bowden A The systems biology markup language (SBML): a medium for representation and exchange of biochemical network models Bioinformatics 2003 19 4 524 531 10.1093/bioinformatics/btg015 12611808

[B6] Wang XS Gorlitsky R Almeida JS From XML to RDF: how semantic web technologies will change the design of 'omic' standards Nature Biotechnology 2005 23 9 1099 1103 10.1038/nbt1139 16151403

[B7] Kell DB Mendes P The markup is the model: reasoning about systems biology models in the Semantic Web era Journal of Theoretical Biology 2008 252 3 538 543 10.1016/j.jtbi.2007.10.023 18054049

[B8] Le Novere N Finney A Hucka M Bhalla US Campagne F Collado-Vides J Crampin EJ Halstead M Klipp E Mendes P Minimum information requested in the annotation of biochemical models (MIRIAM) Nature Biotechnology 2005 23 12 1509 1515 10.1038/nbt1156 16333295

[B9] Laibe C Le Novere N MIRIAM resources: tools to generate and resolve robust cross-references in Systems Biology BMC Systems Biology 2007 1 58 10.1186/1752-0509-1-58 18078503 PMC2259379

[B10] Apweiler R Martin MJ O'Donovan C Magrane M Alam-Faruque Y Antunes R Barrell D Bely B Bingley M Binns D The Universal Protein Resource (UniProt) in 2010 Nucleic Acids Research 2010 38 D142 D148 10.1093/nar/gkp846 19843607 PMC2808944

[B11] Weng S Dong Q Balakrishnan R Christie K Costanzo M Dolinski K Dwight SS Engel S Fisk DG Hong E Saccharomyces Genome Database (SGD) provides biochemical and structural information for budding yeast proteins Nucleic Acids Research 2003 31 1 216 218 10.1093/nar/gkg054 12519985 PMC165501

[B12] PubMed http://www.ncbi.nlm.nih.gov/pubmed/

[B13] de Matos P Alcantara R Dekker A Ennis M Hastings J Haug K Spiteri I Turner S Steinbeck C Chemical Entities of Biological Interest: An update Nucleic Acids Research 2009 38 D249 254 10.1093/nar/gkp886 19854951 PMC2808869

[B14] YeastNet: A consensus reconstruction of yeast metabolism http://www.comp-sys-bio.org/yeastnet/

[B15] B-Net: A schema for representing detailed biochemical knowledge http://mendes.vbi.vt.edu/tiki-index.php?page=B-Net

[B16] Mo ML Palsson BØ Herrgård MJ Connecting extracellular metabolomic measurements to intracellular flux states in yeast BMC Systems Biology 2009 3 37 10.1186/1752-0509-3-37 19321003 PMC2679711

[B17] Nookaew I Jewett MC Meechai A Thammarongtham C Laoteng K Cheevadhanarak S Nielsen J Bhumiratana S The genome-scale metabolic model iIN800 of Saccharomyces cerevisiae and its validation: a scaffold to query lipid metabolism BMC Systems Biology 2008 2 71 10.1186/1752-0509-2-71 18687109 PMC2542360

[B18] Heinisch JJ Müller S Schlüter E Jacoby J Rodicio R Investigation of two yeast genes encoding putative isoenzymes of phosphoglycerate mutase Yeast 1998 14 3 203 213 10.1002/(SICI)1097-0061(199802)14:3<203::AID-YEA205>3.0.CO;2-8 9544241

[B19] Ratledge C Cohen Z Microbial and algal oils: Do they have a future for biodiesel or as commodity oils? Lipid Technology 2008 20 7 155 160 10.1002/lite.200800044

[B20] Fahy E Sud M Cotter D Subramaniam S LIPID MAPS online tools for lipid research Nucleic Acids Research 2007 35 W606 612 10.1093/nar/gkm324 17584797 PMC1933166

[B21] Kanehisa M Goto S Hattori M Aoki-Kinoshita KF Itoh M Kawashima S Katayama T Araki M Hirakawa M From genomics to chemical genomics: new developments in KEGG Nucleic Acids Research 2006 34 D354 D357 10.1093/nar/gkj102 16381885 PMC1347464

[B22] Hubbard TJP Aken BL Ayling S Ballester B Beal K Bragin E Brent S Chen Y Clapham P Clarke L Ensembl 2009 Nucleic Acids Research 2009 37 D690 D697 10.1093/nar/gkn828 19033362 PMC2686571

[B23] Mahadevan R Schilling CH The effects of alternate optimal solutions in constraint-based genome-scale metabolic models Metabolic Engineering 2003 5 4 264 276 10.1016/j.ymben.2003.09.002 14642354

[B24] Kell DB Oliver SG Here is the evidence, now what is the hypothesis? The complementary roles of inductive and hypothesis-driven science in the post-genomic era Bioessays 2004 26 1 99 105 10.1002/bies.10385 14696046

[B25] Kauffman KJ Prakash P Edwards JS Advances in flux balance analysis Current Opinion in Biotechnology 2003 14 5 491 496 10.1016/j.copbio.2003.08.001 14580578

[B26] Becker SA Feist AM Mo ML Hannum G Palsson BØ Herrgård MJ Quantitative prediction of cellular metabolism with constraint-based models: the COBRA Toolbox Nature Protocols 2007 2 3 727 738 10.1038/nprot.2007.99 17406635

[B27] Le Novère N Courtot M Laibe C Adding semantics in kinetics models of biochemical pathways Proceedings of the 2nd International Symposium on experimental standard conditions of enzyme characterizations: 2006 2006 Rüdesheim, Germany Beilstein Institut 137 153

[B28] Ashburner M Ball CA Blake JA Botstein D Butler H Cherry JM Davis AP Dolinski K Dwight SS Eppig JT Gene Ontology: tool for the unification of biology Nature Genetics 2000 25 1 25 29 10.1038/75556 10802651 PMC3037419

[B29] Bornstein BJ Keating SM Jouraku A Hucka M LibSBML: An API library for SBML Bioinformatics 2008 24 6 880 881 10.1093/bioinformatics/btn051 18252737 PMC2517632

[B30] Makhorin A GNU Linear Programming Kit 2001 Moscow: Moscow Aviation Institute

[B31] Giaever G Chu AM Ni L Connelly C Riles L Véronneau S Dow S Lucau-Danila A Anderson K André B Functional profiling of the Saccharomyces cerevisiae genome Nature 2002 418 6896 387 391 10.1038/nature00935 12140549

[B32] Snitkin ES Dudley AM Janse DM Wong K Church GM Segrè D Model-driven analysis of experimentally determined growth phenotypes for 465 yeast gene deletion mutants under 16 different conditions Genome Biology 2008 9 9 R140 10.1186/gb-2008-9-9-r140 18808699 PMC2592718

[B33] SGD project: ACS1/YAL054C http://www.yeastgenome.org/cgi-bin/locus.fpl?dbid=S000000050

[B34] van den Berg MA de Jong-Gubbels P Kortland CJ van Dijken JP Pronk JT Steensma HY The two acetyl-coenzyme A synthetases of Saccharomyces cerevisiae differ with respect to kinetic properties and transcriptional regulation Journal of Biological Chemistry 1996 271 46 28953 28959 10.1074/jbc.271.46.28953 8910545

[B35] King RD Rowland J Oliver SG Young M Aubrey W Byrne E Liakata M Markham M Pir P Soldatova LN The Automation of Science Science 2009 324 5923 85 89 10.1126/science.1165620 19342587

[B36] Aho T Almusa H Matilainen J Larjo A Ruusuvuori P Aho KL Wilhelm T Lähdesmäki H Beyer A Harju M Reconstruction and validation of RefRec: a global model for the yeast molecular interaction network PLoS ONE 5 5 e10662 10.1371/journal.pone.0010662 20498836 PMC2871048

[B37] Hult K Berglund P Enzyme promiscuity: mechanism and applications Trends in Biotechnology 2007 25 5 231 238 10.1016/j.tibtech.2007.03.002 17379338

[B38] Nobeli I Favia AD Thornton JM Protein promiscuity and its implications for biotechnology Nature Biotechnology 2009 27 2 157 167 10.1038/nbt1519 19204698

